# Supplementary material for: Cryo-EM structure of the benzodiazepine-sensitive α1β1γ2S tri-heteromeric GABAA receptor in complex with GABA
Source: eLife. 2018 Jul 25;7:e39383. doi: 10.7554/eLife.39383 (PMC6086659; doi:10.7554/eLife.39383)
Supplement: Supplementary file 1. [file elife-39383-supp1.docx]

| Ligands | GABA | |
| --- | --- | --- |
| **Data collection processing** | Whole map | ECD |
| Microscope | Titan Krios | |
| Voltage(kV) | 300 | |
| Defocus range (μm) | 1.2-2.5 | |
| Exposure time (s) | 40s | |
| Camera | Falcon3 | |
| Dose rate (*e^-^*/Å^2^/s) | 0.6 | |
| Number of frames | 200 | |
| Pixel size (Å) | 0.649 | |
| Particles processed | 216543 | |
| Particles refined | 49147 68229 | |
| Resolution (Å)^$^ | 3.8 | 3.1 |
| **Model Statistics** |  | |
| Number of atoms | 12837 | |
| Protein | 12813 | |
| Ligand | 24 | |
| r.m.s. deviations |  | |
| Bond length (Å) | 0.004 | 0.005 |
| Bond angle (˚) | 0.876 | 0.899 |
| Ramachandran plot |  | |
| Favored (%) | 94.28 | 94.31 |
| Allowed (%) | 5.72 | 5.69 |
| Disallowed (%) | 0.00 | 0.00 |
| Clash score | 4.94 | 4.73 |

$ indicates the resolution reported by Relion without postprocessing

**Supplementary File 1**
