# Supplementary material for: Cryo-EM structure of the benzodiazepine-sensitive α1β1γ2S tri-heteromeric GABAA receptor in complex with GABA
Source: eLife. 2018 Jul 25;7:e39383. doi: 10.7554/eLife.39383 (PMC6086659; doi:10.7554/eLife.39383)
Supplement: Supplementary file 2. [file elife-39383-supp2.docx]

| **Interface**  **Area(**Å^2^**)** | **αβ** | **βα*** | **α*γ** | **γβ*** | **β*α** |
| --- | --- | --- | --- | --- | --- |
| **S_+_** | 12795 | 12290 | 12318 | 11892 | 12291 |
| **S_-_** | 12290 | 12318 | 11892 | 12291 | 12795 |
| **S_T_** | 21917 | 21669 | 21898 | 21539 | 21885 |
| **S_interface_** | 1584 | 1470 | 1156 | 1322 | 1601 |

Note: The solvent accessible surface area is measured using the script get_area in Pymol. The transmembrane helixes were deleted before measuring the solvent accessible surface area. S_+_ stands for the surface area of the isolated subunit at (+) side, S_-_ for the isolated subunit at (-) side, S_T_ for isolated dimer and S_interface_ for the interface between two subunits. The S_interface_ is finally calculated by (S_+_+ S_-_-S_T_)/2.

**Supplementary File** **2.**
